# Hypertension in Cardio-Oncology Clinic: an update on etiology, assessment, and management

**DOI:** 10.1186/s40959-023-00197-8

**Published:** 2023-12-12

**Authors:** Amir Askarinejad, Azin Alizadehasl, Amir Ghaffari Jolfayi, Sara Adimi

**Affiliations:** 1 Rajaie Cardiovascular Medical and Research Center, Iran University of Medical Sciences, Tehran, IR Iran; 2 Cardio-Oncology Research Center, Rajaie Cardiovascular Medical and Research Center, Iran University of Medical Sciences, Tehran, Iran

**Keywords:** Cardio-oncology, Hypertension, Vascular endothelial growth factor inhibitor, Tyrosine kinase inhibitors

## Abstract

Hypertension is one of the most common comorbidity and the leading cause of cancer-related death in cancer patients. The prevalence of hypertension in cancer patients is much higher than that of the general population. In the older population of cancer patients, specific cancer treatments such as new tyrosine kinase inhibitors and Vascular endothelial growth factor inhibitor drugs give rise to hypertension in cancer patients; The aim of present study is to provide a detailed discussion etiologies of cancer treatment-induced hypertension and explore the most innovative diagnostic and management approaches. This review will address the optimal approach to hypertension treatment, covering treatment initiation thresholds, targets, and the selection of anti-hypertensive agents. The lack of evidence in recent guidelines for managing cardiovascular toxicities in cancer patients can create uncertainty in clinicians' therapeutic and clinical decisions. This review aims to enhance our understanding of hypertension etiology in cancer patients and provide a practical guide to current treatment approaches.

## Background

Improvements in early diagnosis and novel cancer treatments have increased the survival rate of cancer patients [1]. Decreased cancer mortality due to novel cancer therapies resulting from novel cancer therapies has led to an increase in short- and long-term cardiovascular complications in these patients [2]. Cardiovascular disease is one of the most common cause of mortality and morbidity in cancer patients [3]. Among cancer patients, cardiovascular adverse events such as heart failure, coronary artery disease, valvular heart disease, and hypertension are prevalent [4, 5]. Previous studies have shown that hypertension is associated with an increased risk of developing certain malignancies, and hypertensive patient experienced higher rates of cancer-related mortality [6–8]. In the older population of cancer patients, specific cancer treatments such as new tyrosine kinase-targeting and Vascular endothelial growth factor inhibitor (VEGFi) drugs can lead to hypertension in cancer patients [9, 10]. Certain pieces of evidence in recent guidelines on managing cardiovascular toxicities in cancer patients may create uncertainty in clinicians' therapeutic and clinical decisions [11, 12]. Hypertension management in cardio-oncology presents significant challenges [13]. Our review aims to provide an updated understanding of the etiology and management of hypertension in cancer patients and offer a practical guide to current treatment approaches. Given the high prevalence of various cancer patients, hypertension management in oncology is applicable not only to cardio-oncologists but would be an essential part of practice among oncologists, general cardiologists, and primary care physicians in the near future. This review has the potential to be a reference guide for clinicians who care for cancer patients with hypertension.

## Etiologies of cancer treatment-induced Hypertension

### Vascular endothelial growth factor inhibitors

Vascular endothelial growth factor inhibitors (VEGFi) drugs are effective and safe components of the anti-cancer treatment for patients with solid malignancy tumors [14]. Next, we will discuss the mention most commonly used VEGFi drugs in cancer treatment. Bevacizumab (Avastin) is utilized in the treatment of various cancers, including colorectal, lung, kidney, and ovarian cancer [15]. Sorafenib (Nexavar) is also commonly used to treat Renal cell carcinoma, hepatocellular carcinoma, and advanced thyroid carcinoma [16]. Sunitinib (Sutent) is another drug used to treat kidney cancer (renal cell carcinoma) and gastrointestinal stromal tumors (GISTs) [17, 18].

Notably, VEGFi increases blood pressure in almost all patients [19]. VEGFi-induced hypertension is dose-dependent and is associated with these drugs' anti-cancer effects [20]. VEGFi increases blood pressure by increasing endothelin-1(ET-1), reducing NO bioavailability, and increasing salt sensitivity by activating the renal epithelial sodium channels(ENaC) [20]. Moreover, some new findings indicate the critical roles of prostacyclin and endothelial microparticles in VEGFi-induced hypertension [21–25].

The primary mechanism of VEGFi-induced hypertension is increased ET-1, leading to vasoconstriction and hypertension [26, 27]. VEGFi increases ET-1 with several mechanisms, including the inactivation of ET_B_ receptors, endothelial dysfunction, and producing vasoconstrictor prostanoids [14, 23, 28, 29]. VEGFi decreases Nitric Oxide(NO) by inactivating the endothelial nitric oxide synthase (eNOS) enzyme and subsequently reduces the bioavailability of NO [30]. As mentioned above, increased vasoconstrictors, mainly ET-1 and decreased vasodilators, especially NO, are two key mechanisms of VEGFi-induced hypertension.

The incidence of VEGFi-induced hypertension varies based on differences in genetics, dosage, and duration of treatment [13]. Axitinib and Sorafenib induce hypertension in 40.4% and 29.0% of patients with renal cell carcinoma, respectively [31]. Recent studies have reported the incidence and relative risk of VEGFi-induced hypertension to be ranging from 4% to 84% and 3 to 9%, respectively [13, 20].

### Immunotherapeutic agents

#### Mammalian target rapamycin (mTOR) inhibitors

Mammalian target rapamycin (mTOR) inhibitors are used as an effective anti-cancer treatment in various solid organ neoplasms like pancreatic neuroendocrine tumors, mantle cell lymphoma, and renal cancer [32, 33]. mTOR inhibitors may induce hypertension through various mechanisms, including increased oxidative stress and sympathetic activation, leading to afferent arteriolar vasoconstriction and hypertension [34].

Everolimus (mTOR inhibitor) in combination with lenvatinib causes hypertension in 42% of metastatic renal cell carcinoma patients [35]. Notably, the incidence of hypertension with everolimus and sirolimus ranges between 17%-30% and 21%-38%, respectively. Hypertension is a common side effect of mTOR inhibitors, whereas some clinical trials demonstrate no significant hypertension increase compared to comparator agents [34, 36, 37].

#### Proteasome inhibitor

Dysregulation of The ubiquitin-proteasome system by proteasome inhibitors like bortezomib and carfilzomib has been tested in different cancers, such as multiple myeloma [38]. Proteasome inhibition in cardiomyocytes and smooth muscle endothelium, resulting in dysregulated NO hemostasis and vasoconstriction are possible mechanisms of hypertension induced by these drugs [39, 40]. Carfilzomib is strongly associated with increased cardiovascular adverse events, including heart failure, hypertension, cardiac ischemia, arrhythmia, and cardiac arrest [41]. Carfilzomib, a first-generation proteasome inhibitor, is associated with the risk of hypertension in multiple myeloma patients (HR= 3.33, *p* < 0.0001) [42].

### Traditional chemotherapeutic agents

Alkylating agents, the earliest drugs used in cancer treatment, work by ultimately creating cross-linkage between two DNA strands, leading to cell death [43]. These drugs are frequently used in the treatment of leukemia, lymphoma, head and neck cancers, and genitourinary cancers [44]. The connection between Alkylating agents and hypertension remains unclear because they are usually used in combination with other drugs for cancer treatment [45]. Cyclophosphamide has a wide range of complications, such as nephrotoxicity, endothelial injury, and abnormalities in the renin-angiotensin system leading to hypertension [46, 47]. While the mechanisms mentioned above could potentionally render Cyclophosphamide-associated Hypertension possible, it has not yet been recognized as an independent risk factor for hypertension in cancer patients. Hypertension was reported in pediatric cancer patients who were treated with ifosfamide [48].

Recent studies do not indicate hypertension as one of the complications in cancer treatment field [49]. Notably, in the meta-analysis of Zhang et al., Hypertension (OR: 2.95, 95%CI: 1.75-4.97, *p* < 0.0001) is one of the risk factors of anthracycline-induced cardiotoxicity [50].

Since the first time cisplatin was used for cancer treatment, platinum-based cancer therapy drugs have been one of the most popular and widespread of their kind for cancer treatment [51]. Platinum-based drugs are used in the treatment of lymphomas, sarcomas, breast, colorectal, germ cell, ovarian, lung, gastro-oesophageal, and bladder cancers [52]. In a study by Herradón et al., the administration of cisplatin for five weeks caused a decrease in systolic and diastolic blood pressure in rats [53]. After ten weeks of treatment, blood pressure decreased significantly after cisplatin- and bleomycin-containing chemotherapy for testicular cancer [54]. Notably, after a median follow-up time of 11.2 years in patients with testicular cancer, the ones treated with cisplatin had higher systolic and diastolic blood pressure levels than those treated with surgery [55]. Therefore, it can be concluded that cisplatin treatment may cause a decrease in blood pressure in early stages of treatment, but, in the long run, it will bring about an upsurge in systolic and diastolic blood pressure.

### BCR-ABL tyrosine-kinase inhibitors (TKIs)

BCR-ABL tyrosine kinase inhibitors (TKIs) are a type of targeted therapy agents used in cancer treatment, particularly in chronic myeloid leukemia (CML). BCR-ABL TKIs block the function of the BCR-ABL1 protein, causing CML cells to die. The first-line of treatment for CML is BCR-ABL TKI therapy, which includes imatinib mesylate, dasatinib, nilotinib, and bosutinib. BCR-ABL TKIs have been developed as a targeted treatment for BCR-ABL1 kinase activity suppression and are used to treat various malignancies. Second-generation BCR-ABL TKIs have been developed to overcome mutations. Drug resistance is the main challenge in BCR-ABL TKI therapy, and designing treatment strategies targeting epigenetic pathways is a potential solution [56, 57].

Limited data available suggests that BCR-ABL tyrosine-kinase inhibitors (TKIs) are associated with multiple cardiovascular and pulmonary adverse events, including pulmonary hypertension, potentially attributable to the fact that the inhibition of the tyrosine kinase BCR-ABL1 is not specific to cancer cells, but rather can affect healthy cells. Renovascular hypertension has been reported as a rare side effect of BCR-ABL TKIs that inhibits vascular endothelial growth factors. However, edema and fluid retention are reported as more common side effects of BCR-ABL TKIs. Recent evidence suggests that tyrosine kinase has a particular role in cardiovascular calcification, specifically the calcification of heart vessels and valves [58, 59].

### BRAF and MEK inhibitors

BRAF and MEK inhibitors are targeted therapies used in cancer treatment, targeting the MAPK signaling pathway. Trametinib was the first FDA-approved MEK inhibitor for cancer therapy which has the property of BRAF/MEK heterodimer breaker and binds to the interface of MEK and BRAF [60]. These groups of drugs are effective in the treatment of various types of cancer, particularly those accociated with RAS or BRAF mutations [61]. BRAF and MEK inhibitors have several side effects, including skin rash, diarrhea, nausea, vomiting, fatigue, and liver toxicity. MEK inhibitors monotherapy or in combination with other targeted drugs harboring the MAPK pathway is becoming a promising strategy for non-small cell lung cancer (NSCLC) patients with BRAF or KRAS mutations [62]. It is noteworthy to mention that 19.5% of hypertension incidence is observed in patients who have used BRAF and MEK inhibitors [63]. In a meta-analysis on the incidence of treatment-related adverse events of BRAF and MEK inhibitors, for patients receiving Dabrafenib + Trametinib, the predominant grade 3 or higher adverse events were pyrexia, rash, and hypertension, collectively accounting for 6% of cases. Also individuals undergoing Encorafenib + Binimetinib regimen encountered incidences of rash and hypertension, amounting to 6% [64].

### Vinca alkaloids

Vinca alkaloids are effective microtubule-targeting agents used in treating hematological and lymphatic neoplasms [65]. Recent studies suggest Vinca Alkaloids are associated with cardiovascular adverse events, such as hypertension [66, 67]. The mechanism of Vinca Alkaloids-induced hypertension is unclear, however, mitosis-mediated inhibition of endothelial cell proliferation and endothelial cell caspase-mediated apoptosis are the possible routes [44]. Considering Vinca Alkaloids are usually used in combination with other chemotherapy agents, the specific effects of these drugs on patients' blood pressure are not well defined.

### Endocrine therapy

Recent improvements in understanding how tumors evolve during treatment with endocrine agents have identified changes in gene expression and mutational profiles in the primary cancer cells as well as in circulating tumor cells. Endocrine therapy is a standard treatment for hormone receptor-positive breast cancer [68].

Anti-androgen therapy, including Cyproterone acetate, Flutamide, Bicalutamide, and Enzalutamide, is adjuvant chemotherapy used to treat different cancer types, such as prostate, breast, kidney, and ovarian cancer [69]. Abiraterone and enzalutamide are novel anti-cancer agents used in the treatment of prostate cancer. Based on a meta-analysis of 7 articles, Abiraterone has induced hypertension in 20% of patients. It was associated with an increased risk of cardiotoxicity [70]. Notably, enzalutamide was associated with increased risks of any grade (RR = 2.66, 95% CI = 1.93-3.66) and severe grade hypertension (RR = 2.79, 95% CI = 1.86-4.18) [71]. Anti-androgen agents cause hypertension by blocking the cytochrome P450 17A1, leading to a decrease in androgen synthesis and an increase in ACTH, resulting in increased mineralocorticoid production. Increased production of mineralocorticoids will lead to hypertension [72].

### Adjuvant therapies

Corticosteroids are used in cancer treatment as adjuvant therapy for pain alleviation or as an antineoplastic agent in treating brain tumors [73, 74]. Corticosteroids such as hydrocortisone, methylprednisone, and prednisone cause dose-dependent hypertension because of their mineralocorticoid effect, especially at high doses [75]. They cause salt and water retention and increase sensitivity to other vasoconstrictive drugs, eventually leading to hypertension [13].

Calcineurin inhibitors like cyclosporine and tacrolimus are essential immunosuppressive drugs used in oncology, almost as adjuvant therapy [76, 77]. They cause salt and water retention and increase sensitivity to other vasoconstrictive drugs, eventually leading to hypertension [78]. They suppressed the transcription of IL-2 and several other cytokines in T lymphocytes [79].

Hypertension is a common side effect of erythropoietin-stimulating agents in healthy individuals, especially in patients with chronic kidney disease [80–82]. Based on a meta-analysis of 52 clinical trials, using erythropoietin-stimulating agents to treat cancer-related anemia causes hypertension and increases adverse events such as mortality [83]. Vasoconstriction of vessels by rising levels of ET-1 and constrictor prostanoids, calcium influx in smooth muscle cells of blood vessels, and anti-natriuresis are underlying mechanisms of erythropoietin-stimulating agents-induced hypertension [84].

30% to 50% of individuals with cancer will experience moderate to severe pain, and non-steroidal anti-inflammatory drugs (NSAIDs) are widely used for chronic pain control in cancer patients [85, 86]. Hypertension is one of the side effects of regular NSAID use because they inhibit the production of prostaglandins; a decrease in prostaglandins I2 and E2 results in vasoconstriction and sodium retention, provoking hypertension [87, 88].

### Radiotherapy

Head and neck radiotherapy may subsequently cause baroreflex failure, leading to resistant hypertension and hypertension crisis [89, 90]. Conversely, in an analysis of 19 patients undergoing head and neck radiation, the systolic and diastolic blood pressure was significantly reduced 90 days after surgery [91].

The baroreceptors regulate blood pressure by transmitting signals to the central nervous system, altering peripheral vascular resistance and cardiac output. Development of labile or paroxysmal hypertension in some individuals after receiving radiation treatment were observed in some studies [92]. Labile hypertension is defined as occasional asymptomatic blood pressure variations, usually attributable to emotional distress [93]. Paroxysmal hypertension is characterized by a sudden rise of blood pressure, passing over 200/110 mmHg, combined with an abrupt onset of physical symptoms, such as headache, chest discomfort, dizziness, nausea, palpitations, flushing, and sweating, taking anywhere between 10 minutes to several hours [94]. On the other hand, baroreceptors' damage can lead to hypotension. Low blood pressure can occur as a result of peripheral, autonomic nervous, and carotid baroreceptor system damage, leading to orthostatic hypotension, presented by dizziness in an upright position after sitting or lying down for a while [95, 96].

**Fig. 1:**
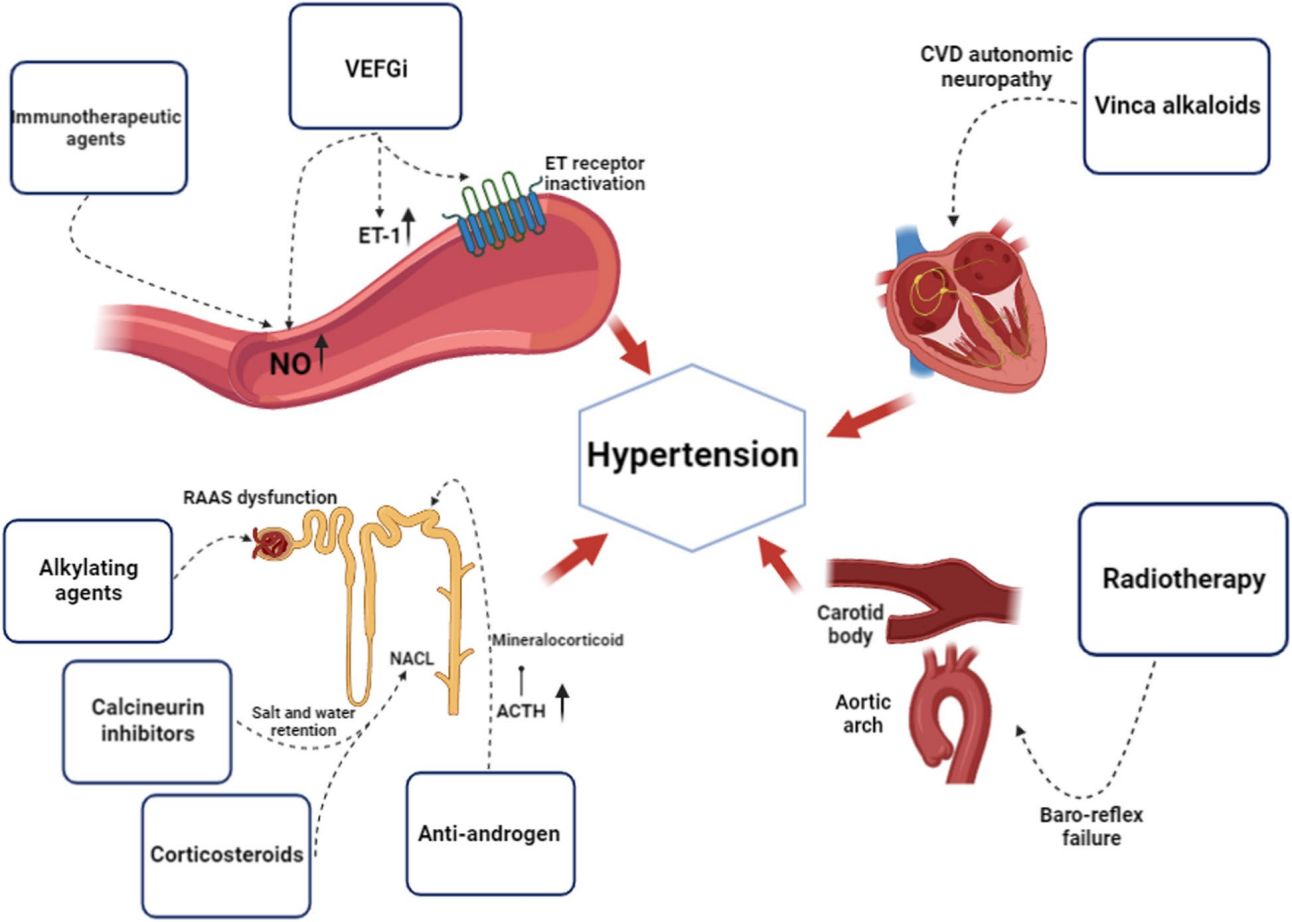
mechanisms of anti-cancer agents in inducing hypertension. Anti-cancer agents induce hypertension through various mechanisms. A better understanding of these mechanisms can help physicians to choose the proper therapeutic strategies for hypertension treatment in cancer patients. ACTH= Adrenocorticotropic Hormone, CVD=Cardiovascular disease, ET= Endothelin, ET-1 = Endothelin-1, NO=Nitric oxide, RAAS= Renin-angiotensin-aldosterone system VEGFi = Vascular endothelial growth factor inhibitor

Radiation nephropathy is a renal injury caused by ionizing radiation [97]. Besides hypertension, hypertensive crisis and encephalopathy may happen as a result of renal injury [97]. Renal artery stenosis is one of the abdominal radiation complications leading to hypertension [98]. Table 1 summarizes the mechanisms of action responsible for hypertension and other side effects of each anti-cancer agents. Figure 1 illustrates these mechanisms in detail.

**Table 1:** Hypertension induced anticancer drugs; mechanism of action and side effects

Medication Group	Mechanism of action leading to hypertension	Drugs	Side effects
Vascular endothelial growth factor inhibitors	• ET-1and vasoconstriction [26, 27]. • through inactivation of ETB receptors, endothelial dysfunction, vasoconstrictor prostanoids • reducing NO bioavailability [30]. • inactivating endothelial eNOS • increasing salt sensitivity [20] • ENaC	Axitinib [31] sorafenib [31]	dose-dependent hypertension [20].
Immune therapeutic agents	Immune checkpoint inhibitors [39, 40, 99] • dysregulated NO hemostasis and vasoconstriction	Bortezomib [38] Carfilzomib [38] Everolimus [35]	Myocarditis [100] arrhythmias, conduction abnormalities [100] pericardial diseases [100] interstitial nephritis glomerulonephritis [99, 101] Acute kidney injury [101] cardiotoxicity [102–104]
	mTOR inhibitors • increased oxidative stress [34] sympathetic activation [34]	Rapamycin	Hyperglycemia [105], hypercholesterolemia [105], hypertriglyceridemia [105]
	CAR-T therapy		fever, flu-like symptoms, low blood pressure, and organ damage [106]
Ubiquitin-proteasome inhibitors [39, 40, 99]	• blocking the β proteolytic subunits of the 20s proteasome	bortezomib and carfilzomib	muscle weakness and tingling [107]
Traditional chemotherapeutic agents	Alkylating agents • creating cross-linkage in DNA strands and cell death [43] • disturbance in renin-angiotensin system [48].	Cyclophosphamide [46, 47] ifosfamide [48] Anthracycline [49] Cisplatin [51].	Nephrotoxicity endothelial injury abnormalities in the renin-angiotensin system [46, 47]
BCR-ABL tyrosine-kinase inhibitors (TKIs)	• inhibiting the development and maturation of monocytes [108]	Nilotinib [109], dasatinib [110], bosutinib [111] and ponatinib [112]	itching and rash, nausea, diarrhea, and tiredness [113]
RAF and MEK inhibitors		vemurafenib and cobimetinib, dabrafenib and trametinib, and encorafenib and binimetinib	rash, diarrhea, peripheral edema, fatigue, and dermatitis acneiform [114]
Vinca alkaloids	• Mitosis-mediated inhibition of endothelial cell proliferation [44]. • Endothelial cell caspase-mediated apoptosis [44]. • Cardiovascular Autonomic neuropathy [115]	Vinblastine [116] Vincristine [117]	Hypertension Hypotension
Endocrine therapy	**Anti-androgens** • Blocking the cytochrome P450 17A1 • Decrease in androgen synthesis Increase in ACTH • Increase mineralocorticoid production	Cyproterone acetate, Flutamide, Bicalutamide, Enzalutamide [69] Abiraterone, Enzalutamide [70]	Cardiotoxicity [70].
Radiotherapy	Baroreflex failure [89, 90].		hypertensive encephalopathy renal injury [97] Renal artery stenosis resulting from abdominal radiation [98]
Adjuvant therapies			
Corticosteroids	Mineralocorticoid Salt and water retention	Hydrocortisone, methylprednisolone, prednisone	induce dose-dependent hypertension
Calcineurin inhibitors	Salt and water retention	cyclosporine tacrolimus	
Non-steroidal anti-inflammatory drugs (NSAIDs)	Inhibit the production of prostaglandins Decrease in prostaglandins I2 and E2 sodium retention [87, 88].	ibuprofen naproxen diclofenac mefenamic acid etoricoxib	

*ENaC* epithelial sodium channel, *mTOR* Mammalian target rapamycin

## Assessment, diagnosis, and management of cancer treatment-induced hypertension

Based on recent studies, cancer patients are considered at a higher risk of hypertension compared to the general population [6]. Hypertension in cancer patients may sometimes occur just after the initiation of chemotherapies and sometimes, years after; hence, both short and long-term management approaches should be considered [118].

Hypertension is associated with an increased risk of mortality among cancer patients [8]. Recent studies have demonstrated that untreated hypertension is strongly associated with the risk of heart failure during treatments with anthracyclines, ibrutinib, and VEGFi [118–121]. Hypertension is an independent risk factor for coronary artery disease, heart failure, valvular heart disease, and arrhythmias in cancer patients [122].

Therefore, Timely diagnosis and proper management of hypertension in cancer patients is a substantial issue for increasing the quality of life and decreasing mortality and morbidity in these patients. Risk assessment for cardiovascular toxicity prior to treatment is essential in cancer patients. Blood pressure should be monitored and assessed before the initiation of chemotherapy [123].

### Assessment and diagnosis

2022 ESC Guidelines on cardio-oncology suggest treatment of hypertension in cancer patients based on 2018 ESC/European Society of Hypertension (ESH) Guidelines [12, 124]. Special cofounders for in-office hypertention in cancer patients are pain, anxiety-driven sympathetic overactivity, NSAIDs, or steroids as adjuvant therapies. Also, it has been noted that both white-coat hypertension and masked hypertension are much more prevalent among cancer patients compared to the general population [125]. Based on 2021 European Society of Hypertension practice guidelines, standard condition, posture, measurement frequency, and interval should be considered to minimizing confounders in in-office hypertention [126].

The 2013 ESH guideline recommended only in-office BP measurement for hypertention diagnosis, however, the 2018 ESH guideline suggested out-of-office BP measurements such as Ambulatory Blood Pressure Monitoring (ABPM) and home blood pressure monitoring (HBPM) [124].

**Fig. 2:**
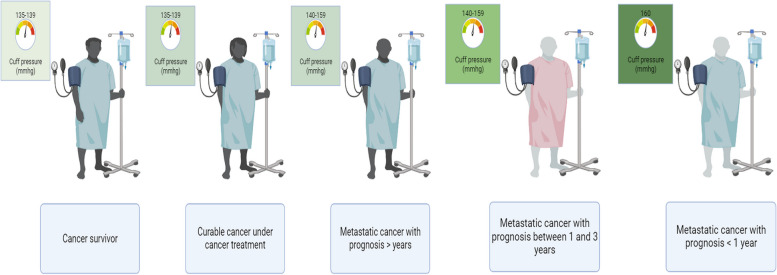
Thresholds for asymptomatic hypertension treatment in cancer patients. The threshold for initiating the hypertension treatment increases as the patient has a worse prognosis. Starting the hypertension treatment at the proper time is crucial in managing hypertension in cancer patients, especially in patients under treatment of VEGFi, because it has been indicated that the efficacy of these drugs is related to blood pressure increase in these patients

**Fig. 3:**
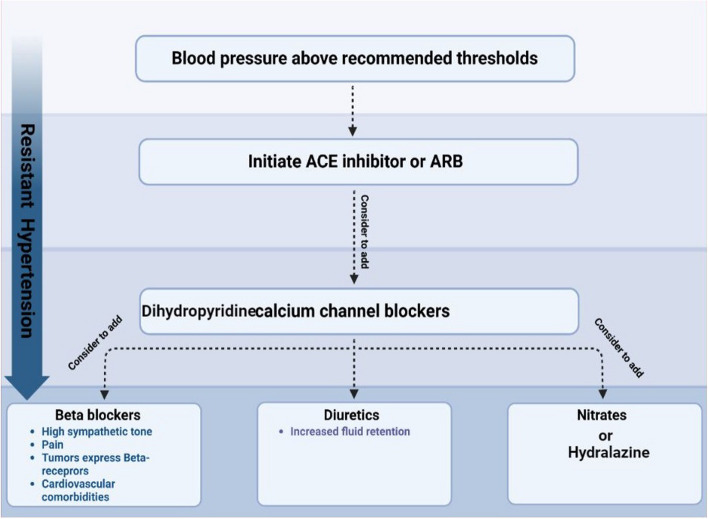
Pharmacological management of hypertension in cancer patients. Choosing the proper anti-hypertensive agent for cancer patients is essential in managing hypertension in these patients. ACE or ARB and CCB are the first-line treatment in hypertension treatment. In patients with resistant hypertention, beta-blockers, diuretics, and nitrates can be added to the therapeutic drugs. Beta-blockers are a good choice if cancer patients have high sympathetic tone, tumors with beta-receptor expression (such as angiosarcoma and multiple myeloma), and other cardiovascular comorbidities

After detecting the initial episodes of increased blood pressure, it is suggested to perform ambulatory blood pressure monitoring for twenty-four hours, but it has its limitations, and it is not feasible for the majority of the patients. Situations in which frequent blood pressure measurements are required over longer periods (during treatment initiation or dose changes in patients receiving anti-cancer treatments) makes the patients susceptible to hypertensive crisis. Worsening of hypertension can occur in a matter of days and can progress to a hypertensive emergency. Hence, home blood pressure monitoring (also known as self-monitoring of blood pressure) with a validated device is an appropriate choice to prevent this situation. This type of monitoring can be done by the patient and is quite feasible. It has less accuracy compared to ambulatory blood pressure monitoring but can be cost-effective in preventing neglected hypertension [63, 127, 128]. It is indicated that cancer patients should take their blood pressure twice a day, once before medication use and bedtime [129]. The threshold for initation of anti-hypertensive agents and how to select the proper anti-hypertensive agents are explained in Figs. 2 and 3. It is recommended to check the blood pressure for a second time 2 weeks after the initiation of the hypertension treatment. If the goals of the treatment are not met, clinicians should take the next step by changing the anti-hypertensive agents, increasing the dose of dihydropyridine CCB or beta-blocker, and perform a sleep study [130].

### Treatment

Treatment thresholds for asymptomatic hypertension in cancer patients are determined by the prognosis, metastasis, and chemotherapy status of the patients. As the prognosis worsens, treatment thresholds for asymptomatic hypertension increase, as shown in Fig. 2.

#### Lifestyle modification as a part of hypertension treatment:

Lifestyle modifications can be an important part of hypertension treatment for cancer patients. The following is an overview of findings that can help manage hypertensive disorders in non-pharmacological treatments.

Various types of anti-cancer medications have been accociated with the developement of hypertension in patients without a prior history of the condition, or with an aggravation of hypertension that was previously under control. Consequently, individuals diagnosed with cancer may be advised to modify their lifestyle in order to assist in managing their hypertension [7].

Modifications in daily life, such as maintaining a healthy weight and exercising regularly, can contribute to reducing blood pressure levels. One may significantly improve their health by following a diet that is low in salt and abundant in fruits, vegetables, and whole grains. Adhering to a consistent schedule of physical activity, limiting alcohol consumption, and cessasion of smoking also have a considerable impact on the prevention of hypertensive disorders [131]. Various classes of medications used for the treatment of cancer have been associated with the development of new-onset hypertension or exacerbation of previously well-controlled hypertension. Management of hypertension in patients on anti-cancer therapy is primarily empirical, with no current trial data supporting specific agents or strategies [63]. Although anti-hypertensive medication use has no consistent evidence of any impact on cancer risk, lifestyle modifications can still be an important part of hypertension treatment in cancer patients [132] Therefore, these modifications are recommended in order to help manage hypertension in cancer patients [133].

#### Pharmacological treatments

Notably, hypertension is an independent risk factor for developing cancer therapy-related cardiac dysfunction; Therefore, Angiotensin-converting enzyme (ACE) inhibitors and Angiotensin receptor blockers (ARBs) are the first choices in the treatment of hypertension in these patients [134]. It has been indicated that using renin-angiotensin system blockers may improve survival in cancer patients. To put it in more detail, in metastatic renal cell carcinoma, patients treated with sunitinib, the use of ACEIs or ARBs was associated with an improved overall survival rates [135–138]. In cases of proteinuria presence, ACEIs or ARBs are recommended as the first line of treatment [139–141].

Dihydropyridine Calcium channel blockers (CCBs) can be used for patients with uncontrolled hypertention despite the treatment with ACE/ ARBs [12, 122]. Notably, hepatotoxicity occurs using some VEGF inhibitors such as Pazopanib, Ponatinib, Regorafenib, Sorafenib, Sunitinib, and Vandetanib. Thus, the use of CCBs should be approached with caution [142].

Non-dihydropyridine calcium channel blockers use cytochrome p450 3A4, possibly leading to decreased metabolism of chemotherapy agents. Therefore, they are not suggested in the treatment of hypertension in cancer patients [143].

The blood pressure target for hypertension treatment in cancer patients is 140/90. Blood pressure targets for patients with chronic kidney disease and diabetes should be 130/80 mmHg [144]. 2022 ESC Guidelines on cardio-oncology suggest a blood pressure target of 140-160/90-100 mmHg for asymptomatic patients suffering from metastatic cancer [12]. Individualized blood pressure targets based on the prognosis of diabetes or kidney disease should be considered in managing hypertension in the cardio-oncology clinics.

If the patient's blood pressure is above 180/110 mmHg, any associated cancer treatment should be withheld tunill the blood pressure is below 160/100 mmHg [3, 12].

Diuretics, nitrates, and beta blockers are suggested for resistant hypertension as first-line Treatments (ACEIs, ARBs, and CCBs ) [12, 145]. Proper anti-hypertensive medication selection depends on various factors, as Fig. 3 demonstrates the approach to hypertension therapy in cancer patients.

#### Patient education

Cancer patients should be educated about the importance of blood pressure control and the potential risks of uncontrolled hypertension, such as target organ damage and cardiovascular adverse events. In this regard, patients should be informed about the causes of hypertension in cancer patients, including various anti-cancer therapies, and the potential side effects of anti-hypertensive drugs. The caregiver must educate them on which anti-cancer therapies have potential risks for developing malignant hypertension [7, 63, 131, 133, 144]. Patients should be educated about lifestyle modifications that can help lower blood pressure, such as weight loss, regular exercise, a healthy diet, and stress reduction. They need to be advised to monitor their blood pressure regularly at home and keep a record of their readings to share with their healthcare providers [7, 92].

#### Patient adherence to treatment

Patients should be encouraged to take their anti-hypertensive medications as prescribed and not to skip doses or discontinue them without consulting with their healthcare providers. Patients should be informed about the potential side effects of anti-hypertensive drugs and how to manage them [7, 144]. Also, they have to be educated to report any symptoms of uncontrolled hypertension to their healthcare providers, such as headache, chest pain, shortness of breath, or vision changes, immediately [131] . They need to be reminded to attend regular follow-up appointments with their physicians to monitor their blood pressure and adjust their treatment if necessary [7, 63, 131, 133, 144].

## Summary and conclusion

**Fig. 4:**
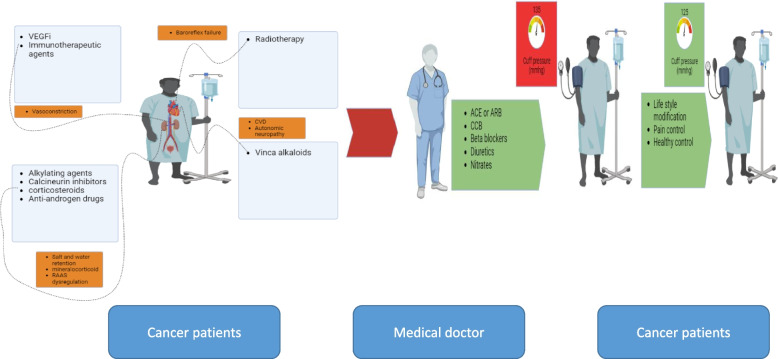
Etiology, Assessment, Diagnosis, and Management of Hypertension in Cancer patients. Anti-cancer agents induce hypertension by different mechanisms, including vasoconstriction, salt and water retention, RAAS dysfunction, baroreflex failure and other mechanisms illustrated in the figure. Physicians should initiate the treatment at the right time by choosing the proper anti-hypertension drug

Hypertension is a common cardiovascular complication in cancer patients, especially those who are treated with angiogenesis inhibitors. Chemotherapeutic agents, Radiation, Adjuvant therapies, and other causes such as pain, renal dysfunction, alcohol consumption, and even untreated sleep apnea cause hypertension in cancer patients. The burden of hypertention in cancer patients is much higher than the general population. Therefore, blood pressure assessment before chemotherapy initiation, timely diagnosis, and appropriate treatment are critical in these patients. In this review, we focused on the details of the appropriate approach for the treatment of hypertension, including thresholds for initiating the treatment, targets, and meticulous selection of anti-hypertensive agents for hypertension management. Figure 4 demonstrates the summary of the etiology, assessment, and management of hypertension in the cardio-oncology clinic.

### Main message

- Hypertension is a common cardiovascular complication with a significant impact on the mortality and morbidity of cancer patients.
- Blood pressure assessment before chemotherapy initiation, timely diagnosis, and appropriate treatment are critical in these patients.
- Further studies are needed to clarify the benefits of hypertension control in cancer patients in detail.

## Abbreviations

AKI: Acute kidney injury
ENaC: Epithelial sodium channel
eNOS: Endothelial nitric oxide synthase
ET-1: Endothelin-1
mTOR: Mammalian target rapamycin
NO: Nitric oxide
TKI: Tyrosine kinase inhibitors
VEGFi: Vascular endothelial growth factor inhibitor
VEGF-TKIs: Vascular endothelial growth factor tyrosine kinase inhibitors
